# HMGB1 Translocation is Associated with Tumor-Associated Myeloid Cells and Involved in the Progression of Fibroblastic Sarcoma

**DOI:** 10.3389/pore.2021.608582

**Published:** 2021-03-31

**Authors:** Huoying Chen, Xiaoying Lin, Hongbo Liu, Cheng Huang, Rong Li, Jie Ai, Jiaxue Wei, Shengjun Xiao

**Affiliations:** ^1^ Prenatal Diagnosis Center, Guangdong Second Provincial General Hospital, Guangdong Provincial Emergency Hospital, Guangzhou, China; ^2^ Department of Laboratory Medicine, The Second Affiliated Hospital of Guilin Medical University, Guilin, China; ^3^ Department of Pathology, The Second Affiliated Hospital of Guilin Medical University, Guilin, China

**Keywords:** high-mobility group box 1 protein, translocation, sarcoma, tumor-associated macrophage, myeloid cell

## Abstract

The morphological variability and genetic complexity of fibroblastic sarcoma makes its diagnosis and treatment a challenge. High-mobility group box 1 protein (HMGB1), which functions as a DNA chaperone and a prototypical damage-associated molecular pattern, plays a paradoxical role in cancer. However, the expression pattern and role of HMGB1 in fibroblastic sarcomas is ill defined. By immunostaining of 95 tissue microarray cores of fibroblastic sarcomas, HMGB1 was found to be expressed in most tumor tissues. Nuclear HMGB1 translocation to cytoplasm was observed both in tumor cells and vascular endothelial cells. A visible number of tumor-associated myeloid cells including CD68^+^ and CD163^+^ macrophages and CD33^+^ myeloid cells were also detected in most tumor tissues. HMGB1 translocation was not only associated with CD68, CD163, and CD33 density, but also with disease progression. These results imply that HMGB1, an important regulator of the tumor microenvironment, is associated with tumor-associated myeloid cells and involved in the progression of fibroblastic sarcomas; HMGB1 may serve as a promising prognostic biomarker and a potential therapeutic target for fibroblastic sarcoma.

## Introduction

Fibroblastic sarcoma is a common panel of soft tissue sarcoma that accounts for approximately 14% of all sarcomas [1] and 12% of pediatric soft tissue tumors [2]. Due to morphologic variability and genetic complexity, the diagnosis and treatment of fibroblastic sarcoma remains a challenge. According to the new 2013 World Health Organization classification, intermediate and malignant fibroblastic sarcoma includes dermatofibrosarcoma protuberans (DFSP), adult-type fibrosarcoma (ATFS), myxofibrosarcoma (MFS) and other histologic subtypes with recurrent cytogenetic or molecular genetic abnormalities [3]. At present, like other sarcomas, the first-line treatment strategy of fibroblastic sarcoma is mainly the combination of surgery, chemotherapy, radiotherapy and other systemic treatment [4]. However, there are limited effective therapy options for treatment as failure often results from local recurrence and distant metastasis [5]. Although new chemotherapeutic drugs such as aldoxorubicin [6], amrubicin [7] and eribulin [8], as well as immune checkpoint blockade agents such as ipilimumab [9] and pembrolizumab [10] for advanced sarcoma are evolving, their approvals are limited to some select histologic subtypes with improved outcomes. Therefore, studies on exploring novel biomarkers involved in the progression of fibroblastic sarcoma and their potential as therapeutic targets are still necessary.

High-mobility group box 1 protein (HMGB1), which performs dual functions as a highly conserved chromosomal protein that enhances transcription and is a crucial cytokine that mediates the response to infection, injury and inflammation, has been reported to play a paradoxical role in cancer [11, 12]. During tumor development and in cancer therapy, extracellular HMGB1 can not only contribute to tumorigenesis but also can stimulate anti-tumor immune responses thought binding to various receptors on different types of cells, such as receptor for advanced glycation end products (RAGE), toll-like receptors (TLRs), chemokine (C–X–C motif) receptor 4 (CXCR4), and T cell immunoglobulin mucin 3 (TIM3) [12–15]. It has been reported that HMGB1 promotes human embryonic lung fibroblast proliferation and extracellular matrix production [16], induces synovial fibroblasts angiogenesis via hypoxia-inducible factor (HIF)-1α activation [17], and induces cardiac fibroblasts migration via CXCR4 in a chemokine (C–X–C motif) ligand 12 (CXCL12) -independent manner [18], which implies that HMGB1 may be involved in the pathogenesis and progression of fibroblastic tumors.

There have been several studies on the role of HMGB1 in fibrosarcoma cells *in vitro*. HMGB1-RAGE signals have been reported to exacerbate the malignant phenotype of HT1080 human fibrosarcoma cell lines [19]. During chemotherapy or radiotherapy, HMGB1 released by dying MCA205 fibrosarcoma cells activated tumor antigen-specific T-cell immunity through acting on TLR4 expressed by antigen presenting cells [20]. Tumor-associated myeloid cells represented by tumor-associated macrophages (TAMs) and myeloid-derived suppressor cells (MDSCs) express multiple receptors of HMGB1 and play an important role in supporting cancer growth and survival, angiogenesis, metastasis as well as immunosuppression [21]. The action of HMGB1 on tumor-associated myeloid cells and their impact on the progression of fibroblastic sarcoma naturally attract attention.

However, no additional studies have focused on the expression and possible role of HMGB1 in fibroblastic sarcomas. Therefore, this study aimed to explore the expression pattern and role of HMGB1 in fibroblastic sarcomas, as well as its relationship with tumor-associated myeloid cells, hoping to provide clues as to whether HMGB1 can be a potential therapeutic target for the disease.

Immunostaining used in this study showed that abundant HMGB1 was expressed in most tumor tissues. Cytoplasm-staining revealed that HMGB1 was present both in tumor cells and vascular endothelial cells. A visible group of TAMs and CD33-positive myeloid cells were also observed in most tumor tissues. HMGB1 expression was not only related to disease progression, but also closely related to tumor-associated myeloid cells. This study implies that HMGB1 plays an important role in the progression of fibroblastic sarcoma and may serve as a useful prognostic biomarker and a potential therapeutic target for the disease.

## Materials and Methods

### Ethics Approval

The process of case collection was approved by Ethics Committee of People’s Hospital of Tongxu County (Henan Province, China). Informed consent from patients was not necessary because the specimen were analyzed retrospectively and already belonged to National Human Genetic Resources Sharing Service Platform (Library number: 2005DKA21300) at the time of the analyses.

### Tissue Microarray

Fibroblastic sarcomas used for construction of tissue microarray (TMA) in this study were obtained from the National Human Genetic Resources Sharing Service Platform (Library number: 2005DKA21300). Ninety-five formalin-fixed, paraffin-embedded tissue blocks of fibroblastic sarcomas including DFSP, MFS and ATFS were selected for construction of the TMA, which was generated by Alenabio (Xi’an, China). Hematoxylin and eosin-stained slides from each tissue block were read by a senior consultant pathologist to obtain 1.0 mm-diameter core biopsies of primary fibroblastic sarcomas. The patient characteristics were also collected and summarized in Table 1. However, follow-up information from the patients was unavailable.

**TABLE 1:** Baseline patient characteristics.

Characteristic	*N* (%) (Total *N* = 95)
Age	
<55	77 (81.05)
≥55	18 (18.95)
Sex	
Female	39 (41.05)
Male	56 (58.95)
Tumor site	
Skin	54 (56.84)
Extremity	23 (24.21)
Trunk and neck	18 (18.95)
Histologic diagnosis	
DFSP	54 (56.84)
MFS	12 (12.63)
ATFS	29 (30.53)
TNM staging	
T1aN0M0+T1bN0M0	63 (66.32)
T2aN0M0+T2bN0M0	32 (33.68)
Tumor grade	
G1	55 (57.89)
G2	35 (36.84)
G3	5 (5.26)
AJCC staging	
IA + IB	55 (57.89)
IIA + IIB	38 (40.00)
III	2 (2.11)

Abbreviations: N, Number; TNM, Tumor Node Metastasis; AJCC, American Joint Committee on *Cancer*; DFSP, Dermatofibrosarcoma protuberans; MFS, Myxofibrosarcoma; ATFS, Adult-type fibrosarcoma.

### Immunohistochemistry

Immunohistochemistry (IHC) was performed on TMA slides with the anti-HMGB1 (1:2000; Abcam, Cambridge, MA, United States), anti-Cluster of Differentiation (CD) 163 (1:200; Abcam, Cambridge, MA, United States), anti-CD33 (1:50; R&D Systems, Minneapolis, MN, United States), and anti-CD68 (Ready-to-use antibody, MXB Biotechnologies, Fuzhou, Fujian Province, China) antibodies using a MaxvisionTM2 HRP-Polymer anti-Mouse/Rabbit IHC Kit according to the manufacturer’s instructions (MXB Biotechnologies, Fuzhou, Fujian Province, China). All slides were counterstained with hematoxylin and mounted. Digital images of immunostained TMA slides were acquired using the KF-PRO-005-EX digital pathology slide scanner (KFBIO, Ningbo, Zhejiang Province, China).

### Histological Scoring

The immunostaining score of HMGB1 was divided into total score, nucleus-staining score and cytoplasm-staining score. The nucleus-staining and cytoplasm-staining of HMGB1 were scored on a scale semiquantitatively based on the percentage of positive cells and staining intensity by the following method. Six fields per TMA core at ×400 magnification were randomly selected. The percentages of HMGB1-positive cells with nucleus-staining or cytoplasm-staining were calculated and scored as follows: 1) score 0, <5%; 2) score 1, ≥5% and <25%; 3) score 2, ≥25% and <50%; 4) score 3, ≥50%. And the staining intensity of HMGB1-positive cells with nucleus-staining or cytoplasm-staining were evaluated and scored as follows: 1) score 0, negative; 2) score 1, weak; 3) score 2, moderate; 4) score 3, strong. The nucleus-staining or cytoplasm-staining score of one field was the sum of the percentage of positive cells and the staining intensity scores. The final score of each TMA core was the mean of the six fields, and the total score of HMGB1 was the sum of nucleus-staining and cytoplasm-staining scores. Scoring was performed by a pathologist experienced in scoring tumor biomarkers. And then statistical analysis was performed on HMGB1 total score, nuclear staining score and cytoplasmic staining score separately.

Immunohistochemical markers of immune cells, including CD68, CD163 and CD33, were scored by counting the number of positive-staining cells per TMA core divided by the area of the core to yield a value for cells/mm^2^. Cell counting was performed by two investigators using the Image-Pro Plus 6.0 software (Media Cybernetics, Rockville, MD, United States). Scoring was calculated from the mean of the two independently conducted assessments.

### Statistics

Statistical analyses were performed using SPSS standard version 16.0 (SPSS Inc. Chicago, IL) and GraphPad Prism version 5.0 (GraphPad Software, SanDiego, CA). Comparisons between groups were analyzed by independent sample *t*-test, one-way analysis of variance (ANOVA) followed by Bonferroni’s test or by Dunnett's T3 test (for non-normal data with unequal variances) for quantitative data, or Mann–Whitney *U* test for ordinal data, as appropriate. The correlation between histological score, cell density and tumor grade was determined by Spearman rank test. *p*-values <0.05 were considered statistically significant.

## Results

### The Immunostaining Pattern of HMGB1 in Fibroblastic Sarcomas

In order to clarify the expression pattern of HMGB1 in fibroblastic sarcomas, the staining of HMGB1 were detected by IHC assay in 95 TMA cores of fibroblastic sarcomas, which included 54 cases of DFSP, 12 cases of MFS and 29 cases of ATFS. The total score, nucleus-staining score and cytoplasm-staining score of HMGB1 per TMA core were then evaluated and statistically analyzed.

**FIGURE 1:**
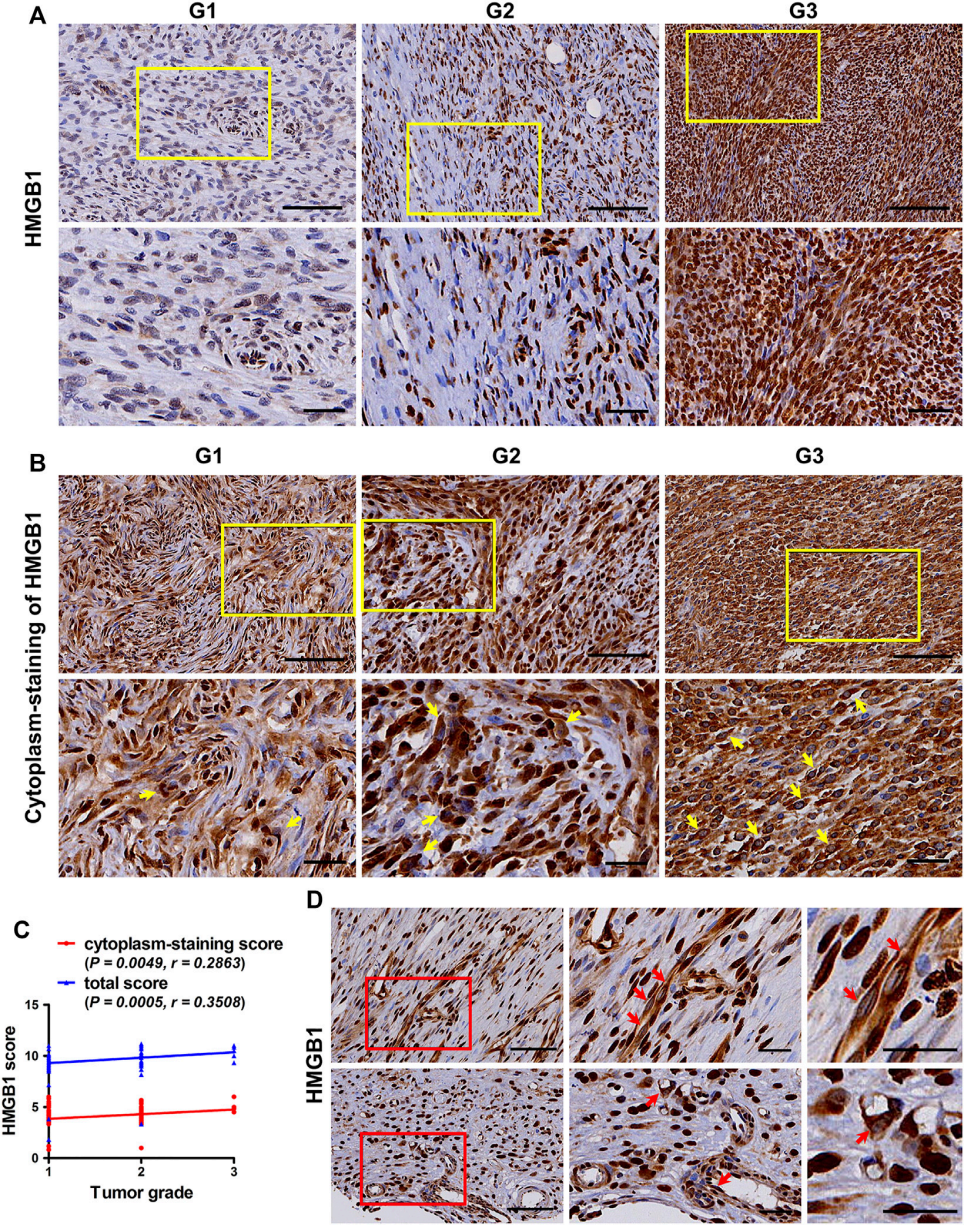
The expression pattern of HMGB1 in fibroblastic sarcomas. **(A)** Representative images of immunostaining of HMGB1 in tumors with different tumor grade. **(B)** Representative images of cytoplasm-staining of HMGB1 in tumors with different tumor grade. HMGB1 cytoplasm-staining-positive tumor cells are indicated by yellow arrow at high magnification, which is framed by yellow rectangles at low magnification. **(C)** Correlation of HMGB1 total score and cytoplasm-staining score with tumor grade in fibroblastic sarcomas. The correlation was determined by Spearman rank correlation test. **(D)** Cytoplasm-staining of HMGB1 in vascular endothelial cells. Cytoplasm-staining HMGB1-positive vascular endothelial cells are indicated by red arrow at high magnification, which is framed by red rectangles at low magnification. Scale bars were 100 µm for low magnification (×200) and 50 µm for high magnification (×400) and for the highest magnification in (**D**).

Under physiological conditions, HMGB1 is usually located in the nucleus. During tumor development and in cancer therapy, HMGB1 is released and plays multiple roles through binding receptors, including RAGE, TRL2/4, TIM3 and CXCR4 [12]. Before releasing, HMGB1 must first be translocated from the nucleus to the cytoplasm [22]. In this study, as shown in Table 2, there was no difference in HMGB1 nucleus-staining score in TMA cores with different tumor staging and grades. However, TMA cores with higher Tumor Node Metastasis (TNM) staging and American Joint Committee on *Cancer* (AJCC) staging, as well as higher tumor grades, had higher HMGB1 cytoplasm-staining score and HMGB1 total score (*p* < 0.05). These results indicated that the difference in HMGB1 expression in tumors with different staging and grades was mainly the difference in HMGB1 cytoplasmic expression. Representative images in Figures 1A,B showed the expression and cytoplasm-staining of HMGB1 in TMA cores with different grades. Cytoplasm-staining of HMGB1 in tumor cells was easily observed in most of the TMA cores. The higher the tumor grade, the easier to observe the cytoplasm staining of HMGB1 in more tumor cells (Figure 1B). Moreover, correlation analysis revealed that HMGB1 cytoplasm-staining score and HMGB1 total score were related to tumor grade (*p* = 0.0049, *r* = 0.2863; *p* = 0.0005, *r* = 0.3508; respectively) (Figure 1C), whereas HMGB1 nucleus-staining score was not related (data not shown). Cytoplasm-staining of HMGB1 in vascular endothelial cells was also observed in some of the TMA cores (Figure 1D). This means that, in addition to tumor cells, HMGB1 could also be released from vascular endothelial cells to play a certain role in fibroblastic sarcomas. These results suggest that HMGB1 derived from tumor cells and vascular endothelial cells may be involved in the progression of fibroblastic sarcomas.

**TABLE 2:** Relationship between HMGB1 score and patient characteristics of fibroblastic sarcomas.

Characteristic	Patients	HMGB1 total score		HMGB1 nucleus-staining score		HMGB1 cytoplasm-staining score	
	N (%)	Mean ± Sd	*p*	Mean ± Sd	*p*	Mean ± Sd	*p*
Total	95 (100)	9.55 ± 1.38		5.48 ± 0.87		4.06 ± 0.97	
Age							
<55	77 (81.05)	9.54 ± 1.33	0.224	5.46 ± 0.87	0.409	4.08 ± 0.98	0.569
≥55	18 (18.95)	9.58 ± 1.63		5.56 ± 0.92		4.02 ± 0.96	
Sex							
Female	39 (41.05)	9.56 ± 1.23	0.831	5.51 ± 0.86	0.332	4.05 ± 1.00	0.498
Male	56 (58.95)	9.54 ± 1.49		5.46 ± 0.89		4.07 ± 0.95	
Tumor site							
Skin	54 (56.84)	9.53 ± 1.19	0.292^a^	5.47 ± 0.87	0.739^a^	4.06 ± 0.90	0.749^a^
Extremity	23 (24.21)	9.57 ± 1.58	0.927^b^	5.43 ± 0.92	0.761^b^	4.14 ± 1.08	0.751^b^
Trunk and neck	18 (18.95)	9.57 ± 1.72	0.413^c^	5.59 ± 0.87	0.394^c^	3.98 ± 1.05	0.911^c^
Histologic diagnosis							
DFSP	54 (56.84)	9.53 ± 1.19	0.867^d^	5.47 ± 0.87	0.986^d^	4.06 ± 0.90	0.920^d^
MFS	12 (12.63)	9.65 ± 0.83	0.387^e^	5.51 ± 0.62	0.581^e^	4.14 ± 0.90	0.854^e^
ATFS	29 (30.53)	9.53 ± 1.86	0.110^f^	5.49 ± 0.99	0.353^f^	4.04 ± 1.13	0.802^f^
TNM staging							
T1aN0M0+T1bN0M0	63 (66.32)	9.30 ± 1.58	0.002	5.36 ± 1.01	0.126	3.94 ± 1.04	0.031
T2aN0M0+T2bN0M0	32 (33.68)	10.02 ± 0.68		5.71 ± 0.45		4.31 ± 0.77	
Tumor grade							
G1	55 (57.89)	9.29 ± 1.40	0.004^g^	5.04 ± 0.97	0.273^g^	3.90 ± 0.94	0.078^g^
G2	35 (36.84)	9.83 ± 1.36	0.402^h^	5.67 ± 0.67	0.197^h^	4.16 ± 0.92	0.008^h^
G3	5 (5.26)	10.33 ± 0.68	0.016^i^	5.07 ± 0.92	0.449^i^	5.27 ± 0.69	0.002^i^
AJCC staging							
IA + IB	55 (57.89)	9.29 ± 1.40	0.001	5.04 ± 0.97	0.449	3.90 ± 0.94	0.016
IIA + IIB + III	40 (42.11)	9.89 ± 1.30		5.60 ± 0.72		4.30 ± 0.96	

Note: a, Skin group vs Extremity group; b, Extremity group vs Trunk and neck group; c, Trunk and neck group vs Skin group; d, DFSP group vs MFS group; e, MFS group vs ATFS group; f, ATFS group vs DFSP group; g, G1 vs G2; h, G2 vs G3; i, G3 vs G1; using Mann-Whitney *U* test.

Abbreviations: N, Number; TNM, Tumor Node Metastasis; AJCC, American Joint Committee on *Cancer*; DFSP, Dermatofibrosarcoma protuberans; MFS, Myxofibrosarcoma; ATFS, Adult-type fibrosarcoma; HMGB1, high-mobility group box 1.

### Immunostaining of CD68, CD163 and CD33 in Fibroblastic Sarcomas

**FIGURE 2:**
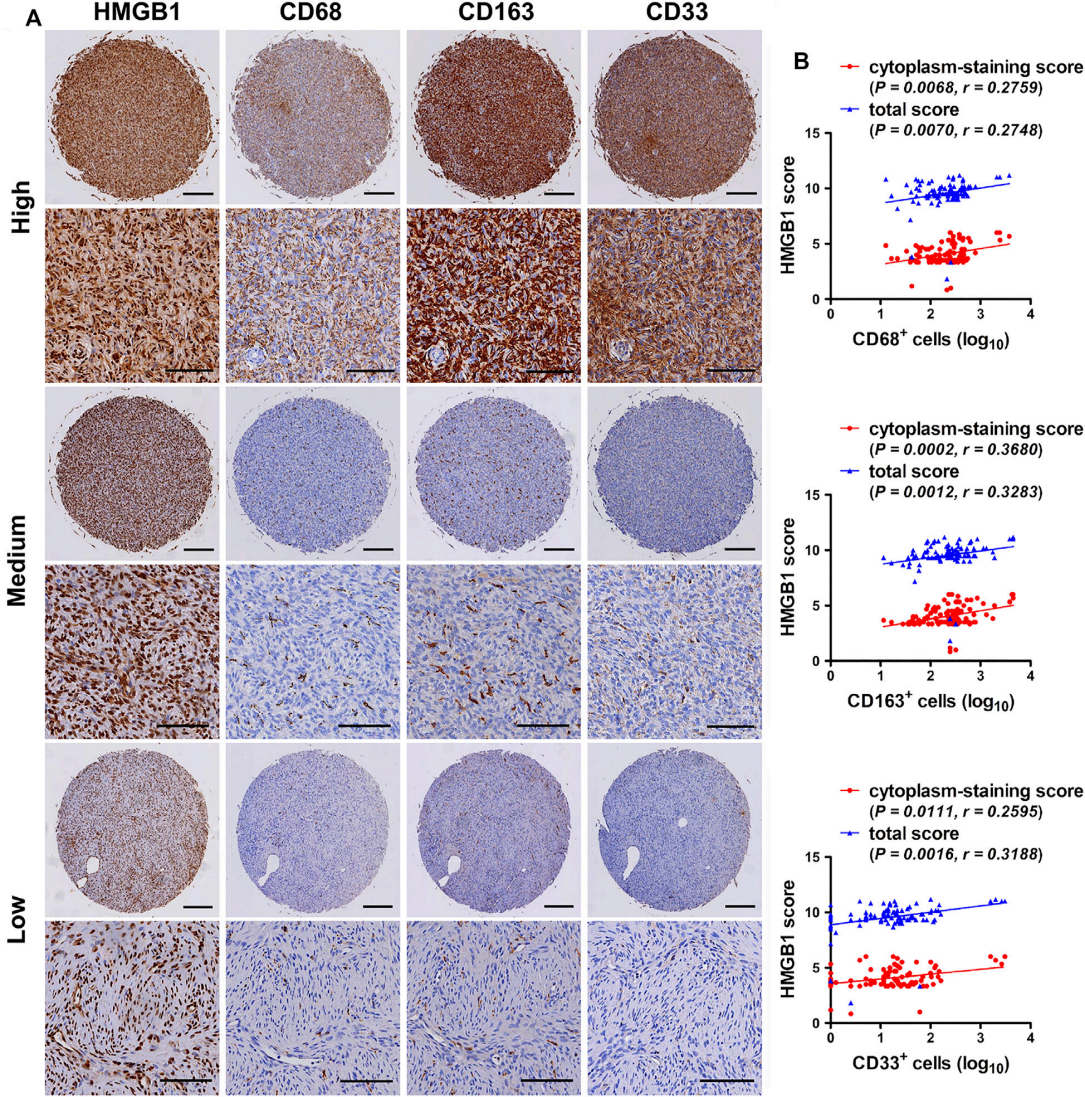
Expression correlation of HMGB1 with CD68, CD163 and CD33 in fibroblastic sarcomas. **(A)** Immunohistochemical staining for HMGB1, CD68, CD163 and CD33-positive cells in fibroblastic sarcomas. Representative images of high, medium or low staining of all Immunohistochemical markers in one same tissue microarray **(**TMA) core are shown. Scale bars were 200 µm for low magnification (×4) and 100 µm for high magnification (×200). **(B)** Correlation of HMGB1 total score and cytoplasm-staining score with CD68, CD163 and CD33 density in fibroblastic sarcomas. Cell density was transformed to log10 scale for statistical analysis and plotting. The correlations were analyzed by Spearman rank correlation test.

In order to clarify the accumulation of TAMs and MDSCs in fibroblastic sarcomas, the desity of CD68 (a pan-macrophage marker), CD163 (a M2-polarized macrophage marker) and CD33 (a myeloid marker that sometimes is used for identification of MDSCs [23]) in 95 TMA cores of fibroblastic sarcomas were also evaluated by immunostaining. As shown in Table 3 and Figure 2A, a considerable number of CD68 [median (range): 243 (13–3,753) cells/mm^2^] and CD163-positive [median (range): 245 (11–4,517) cells/mm^2^] cells were observed in most of the TMA cores. Moreover, TMA cores with higher AJCC staging had higher density of CD163 but not CD68 (*p* < 0.05). Table 3 and Figure 2A also showed that a small group of CD33-positive cells [median (range): 17 (0–3,078) cells/mm^2^] were observed in most of the TMA cores. Although there was a tendency toward a higher CD33 density in tumors with higher TNM staging and AJCC staging, this did not reach statistical significance (Table 3). These results suggest that tumor-associated myeloid cells including TAMs and CD33-positive myeloid cells may play an important role in promoting the development of fibroblastic sarcoma.

**TABLE 3:** Relationship between CD68, CD163 and CD33 density and patient characteristics of fibroblastic sarcomas.

Characteristic	Patients	CD68^+^ cells/mm^2^		CD163^+^ cells/mm^2^		CD33^+^ cells/mm^2^	
	N (%)	Median (range)	*p*	Median (range)	*p*	Median (range)	*p*
Total	95 (100)	243 (13–3,753)		245 (11–4,517)		17 (0–3,078)	
Age							
<55	77 (81.05)	220 (13–3,753)	0.648	231 (11–4,517)	0.833	14 (0–3,078)	0.519
≥55	18 (18.95)	302.5 (33–627)		276.5 (84–1,899)		49.5 (3–161)	
Sex							
Female	39 (41.05)	254 (17–2,419)	0.593	245 (11–4,487)	0.344	14 (0–1,582)	0.277
Male	56 (58.95)	223.5 (13–3,753)		245 (17–4,517)		18 (0–3,078)	
Tumor site							
Skin	54 (56.84)	297.5 (45–2,451)	1.000^a^	260 (17–4,260)	1.000^a^	11 (0–3,078)	1.000^a^
Extremity	23 (24.21)	93 (22–3,753)	1.000^b^	232 (28–4,517)	1.000^b^	20 (0–1,997)	1.000^b^
Trunk and neck	18 (18.95)	138.5 (13–2,419)	1.000^c^	185.5 (11–4,487)	1.000^c^	23.5 (0–1,582)	1.000^c^
Histologic diagnosis							
DFSP	54 (56.84)	297.5 (45–2,451)	1.000^d^	260 (17–4,260)	0.613^d^	11 (0–3,078)	1.000^d^
MFS	12 (12.63)	235.5 (17–2,419)	1.000^e^	392.5 (11–4,487)	0.638^e^	43 (0–1,582)	1.000^e^
ATFS	29 (30.53)	93 (13–3,753)	1.000^f^	208 (28–4,517)	1.000^f^	18 (0–1,997)	1.000^f^
TNM staging							
T1aN0M0+T1bN0M0	63 (66.32)	228 (13–3,753)	0.139	224 (11–4,517)	0.094	11 (0–1,997)	0.120
T2aN0M0+T2bN0M0	32 (33.68)	274.5 (22–2,451)		322 (51–4,487)		32 (0–3,078)	
Tumor grade							
G1	55 (57.89)	242 (17–2,102)	0.730^g^	237 (11–4,260)	0.241^g^	11 (0–3,078)	1.000^g^
G2	35 (36.84)	246 (22–3,753)	0.953^h^	320 (36–4,517)	0.758^h^	23 (0–2,645)	1.000^h^
G3	5 (5.26)	250 (13–2,419)	0.846^i^	232 (121–4,487)	0.550^i^	38 (6–1,581)	0.659^i^
AJCC staging							
IA + IB	55 (57.89)	242 (17–2,102)	0.250	237 (11–4,260)	0.038	11 (0–3,078)	0.241
IIA + IIB + III	40 (42.11)	248 (13–3,753)		315.5 (36–4,517)		23.5 (0–2,645)	

a, Skin group vs Extremity group; b, Extremity group vs Trunk and neck group; c, Trunk and neck group vs Skin group; d, DFSP group vs MFS group; e, MFS group vs ATFS group; f, ATFS group vs DFSP group; g, G1 vs G2; h, G2 vs G3; i, G3 vs G1; using one-way ANOVA followed by Bonferroni’s test or by Dunnett's T3 test if the requirements of normal distribution and equal variance were not fulfilled.

Abbreviations: N, Number; TNM, Tumor Node Metastasis; AJCC, American Joint Committee on *Cancer*; DFSP, Dermatofibrosarcoma protuberans; MFS, Myxofibrosarcoma; ATFS, Adult-type fibrosarcoma; CD, cluster of differentiation.

### Expression Correlation of HMGB1 and CD68, CD163 and CD33 in Fibroblastic Sarcomas

The study explored the relationship between HMGB1 and the density of tumor-associated myeloid cells in fibroblastic sarcomas. Representative images of high, medium or low staining of HMGB1, CD68, CD163 and CD33 in one same TMA core were shown in Figure 2A. Correlation analysis revealed that the density of CD68, CD163 and CD33 was not only positively correlated with the total score of HMGB1, but also with the cytoplasm-staining score of HMGB1 in fibroblastic sarcomas (Figure 2B). However, there was no correlation between HMGB1 nucleus-staining score and CD68, CD163 and CD33 density (data not shown). Taken together, these results indicate that HMGB1 may be released and act on CD68 and CD163-positive M2-polarized macrophages and CD33-positive myeloid cells in fibroblastic sarcomas, and involved in disease progression.

## Discussion

HMGB1 plays a significant role in many cancers and has promising clinical application prospects as a therapeutic target. Several HMGB1-targeting agents have been developed and used in experimental cancer research. These agents include sRAGE [24], HMGB1 neutralizing antibody [25], A box protein [25], ethyl pyruvate [26], quercetin and glycyrrhizin [27], and platinating agents such as cisplatin and oxaliplatin [28]. However, the relationship between HMGB1 and soft tissue sarcoma has received little attention.

This study detected the expression pattern of HMGB1 in several types of fibroblastic sarcomas including DFSP, MFS and ATFS by immunostaining. HMGB1 was found to be expressed in most tumor cells, and a higher expression of HMGB1 was observed in tumor tissues with higher grade and staging. The correlation between the high expression of HMGB1 and tumor grade and staging has also been reported in gastric adenocarcinomas [29] and liver cancer [30]. Furthermore, obvious cytoplasm-staining of HMGB1 was observed both in tumor cells and vascular endothelial cells and positively correlated with tumor grade in fibroblastic sarcoma. Consistently, cytoplasmic expression of HMGB1 was also detected in breast cancer and human renal clear cell cancer and indicated higher tumor grades [31–33]. These results suggest that HMGB1 could be released by tumor cells and vascular endothelial cells and involved in the progression of fibroblastic sarcoma.

The relationship between HMGB1 and tumor-related myeloid cells in fibroblastic sarcoma is unclear. In this study, a visible number of TAMs and CD33-positive myeloid cells were observed in most tumor tissues. It is generally accepted that TAMs possess M2-polarized macrophages which play an immunosuppressive role and promote tumor progression [34, 35]. Although there was similar density of CD68 (staining pan-macrophages) and CD163 (staining M2-polarized macrophages) in fibroblastic sarcoma, only high density of CD163 indicated higher tumor staging. This means that M2-polarized macrophages play a more critical role in the progression of fibroblastic sarcoma. Consistent with this, in some tumor types, including head and neck squamous cell carcinoma and esophageal cancer, a higher density of CD163-positive TAMs is closely correlated to worse clinical courses, whereas no significant association is seen between the density of CD68-positive TAMs and clinical prognosis [36, 37]. Abundant accumulation of CD68 and CD163-positive macrophages in sarcoma is also elucidated in a study involving 24 types of sarcoma including DFSP and MFS. The authors also show that the sarcomas tend toward M2-like macrophage polarization [38], further supporting that M2-polarized macrophages is involved in sarcomas progression. MDSCs, usually labeled in combination with CD33^+^ and other markers, are another important group of tumor-related myeloid cells that accumulate in tumor tissues, promote tumor progression and suppress anti-tumor immune responses [39, 40]. Compared with TAMs, the density of CD33-positive myeloid cells was much lower in fibroblastic sarcoma and not significantly related to disease progression. Higher tumor staging showed a trend of higher density of CD33, which suggests that CD33-positive myeloid cells such as MDSCs also play a part in promoting the development of fibroblastic sarcoma. The correlation between HMGB1 and CD68 has been observed in gastric cancer [41], but the correlation between HMGB1 and CD163 and CD33 has not been reported. In fibroblastic sarcomas, the total score and the cytoplasm-staining score of HMGB1 were positively correlated with CD68, CD163 and CD33 density, indicating that HMGB1 may act on M2-polarized TAMs and CD33-positive myeloid cells in fibroblastic sarcomas and contribute to disease progression. There appears to be a mechanism linking HMGB1 to immunosuppression and myeloid cells including TAMs and CD33-positive myeloid cells in patients with malignant tumors, which needs further studies to uncover.

The present study lacked a survival analysis because follow-up information from the patients was unavailable. Analysis of data from The *Cancer* Genome Atlas (TCGA) and GSE72545 datasets found that MFS patients with higher transcriptional levels of HMGB1 had a poor prognosis. However, CD68, CD163 and CD33 showed no prognostic value for MFS patients (data not shown). In addition, data on DFSP and ATFS in public databases are very limited, and the relationship between the expression of HMGB1, CD68, CD163 and CD33 and the prognosis of DFSP and ATFS remains further research.

Another limitation of this study is that no genetic data were provided on the possible cause of HMGB1 overexpression in fibroblastic sarcomas. By using the cbioportal (http://www.cbioportal.org/), a powerful online website for integrated cancer data analysis, the relationship between HMGB1 mRNA levels and gene mutations, copy-number alterations and methylation in MFS cases from TCGA was analyzed. The results showed that no HMGB1 mutations were detected in patients with MFS. Higher mRNA level of HMGB1 was found in gene gain and diploid group. Moreover, the mRNA level of HMGB1 was significantly negatively correlated with the level of methylation; indicating that high expression of HMGB1 in MFS is related to gene amplification and hypomethylation (data not shown). Similarly, whether this scenario occurs in DFSP and ATFS remains further investigation.

In summary, this study implies that HMGB1 released by tumor cells and vascular endothelial cells may modulate the infiltration, polarization and function of TAMs and CD33-positive myeloid cells, thereby contributing to the progression of fibroblastic sarcomas. Although these conclusions require further confirmation via *in vivo* and *in vitro* experiments, this study provides clues furthering our understanding of the role of HMGB1 in the progression of fibroblastic sarcomas, and suggests that HMGB1 may serve as a useful prognostic biomarker and a potential therapeutic target for the disease.

## Data Availability

The original contributions presented in the study are included in the article/Supplementary Material, further inquiries can be directed to the corresponding author/s.
